# Gemtuzumab ozogamicin and novel antibody-drug conjugates in clinical trials for acute myeloid leukemia

**DOI:** 10.1186/s40364-019-0175-x

**Published:** 2019-10-31

**Authors:** Bo Yu, Delong Liu

**Affiliations:** 1 Department of Medicine, Lincoln Medical Center, Bronx, NY USA; 2 Department of Medicine, New York Medical College and Westchester Medical Center, Valhalla, NY USA; 3 Department of Oncology, The First affiliated Hospital of Zhengzhou University, Zhengzhou, China

**Keywords:** Gemtuzumab ozogamicin, Antibody-drug conjugate, CD33, CD123, CLL1

## Abstract

Targeted agents are increasingly used for the therapy of acute myeloid leukemia (AML). Gemtuzumab ozogamicin (GO) is the first antibody-drug conjugate (ADC) approved for induction therapy of AML. When used in fractionated doses, GO combined with the conventional cytarabine/anthracycline-based induction chemotherapy significantly improves the outcome of previously untreated AML patients. Single-agent GO is effective and safe for AML patient ineligible for intensive chemotherapy. Multiple combination regimens incorporating GO have also been recommended as potential alternative options. In addition, several novel ADCs targeting CD33, CD123 and CLL-1 are currently undergoing preclinical or early clinical investigations. In this review, we summarized the efficacy and limitations of GO as well as novel ADCs for adult AML patients.

## Introduction

The prognosis of AML remains unsatisfactory with a 5-year overall survival (OS) rate of 40% for patients less than 60 years of age and only 10% for elderly patients above the age of 60 [1]. The conventional first-line treatment of AML includes intensive cytarabine/anthracycline-based induction chemotherapy followed by consolidation chemotherapy or allogenic stem cell transplantation (AlloSCT) [2–6]. Best supportive care, low dose cytarabine, and hypomethylating agents (HMA) are often alternative options for elderly patients unfit for intensive chemotherapy [7]. In the past several years, several new targeted agents against FLT3, IDH1, and IDH2 have been approved for AML therapy [8–16]. Novel targeted therapies and immunotherapies against AML are in active clinical development [17–20]. One such example is gemtuzumab ozogamicin (GO), an antibody-drug conjugate (ADC) [21–24].

ADC composes of three essential components: a monoclonal antibody (mAb) that recognizes an antigen target on tumor cells, a cytotoxic molecule often referred to as payload, and a chemical linker that connects the mAb and payload [25–28]. Upon binding to the corresponding antigen on the surface of tumor cells, ADC is internalized first and the linker is hydrolyzed inside of the lysosomes or endosomes, releasing the payloads that lead to cell death by damaging DNA or impeding microtubule assembly. ADC becomes attractive because it enhances targeted killing of tumors while sparing normal tissues, thereby minimizing toxicity. With the improvement of engineering technology, the newest generation of ADC can be generated through site-specific conjugation and has homogenous drug-antibody ratio as well as better stability in circulation [29]. This review summarized the recent updates of GO and other ADCs at different stages of development for AML therapy.

## Gemtuzumab ozogamicin (GO; Mylotarg)

### Clinical pharmacology

GO is a humanized anti-CD33 IgG4 mAb conjugated to a cytotoxic agent N-acetyl gamma calicheamicin via an acid-labile hydrazone linker [29]. CD33 belongs to the family of sialic acid–binding immunoglobulin-like lectins (Siglecs). It is a transmembrane glycoprotein expressed on the surface of immature cells of myelomonocytic lineage and myeloblasts in > 80% of AML patients [30]. High CD33 expression is generally correlated to negative prognostic features [31]. Specifically targeting CD33 does not affect the differentiation of presumably normal CD33-CD34+ myeloid progenitors, allowing for the restoration of normal nonclonal hematopoiesis [32, 33]. Upon binding to CD33, the drug is quickly internalized. Calicheamicin is released in the acidic environment of lysosomes and then binds to DNA, resulting in DNA double-strand breaks and subsequent cell death.

A phase I dose escalation study for GO in CD33+ AML was done with 0.25–9 mg/m^2^ for two doses 14 days apart. The maximum tolerated dose (MTD) was determined to be 9 mg/m^2^, which was recommended for further phase II trials. Infusion reaction, myelosuppression, and bilirubin elevation were generally observed at MTD [34]. Pharmacokinetic studies showed larger than proportional increases in maximum concentration (Cmax) and area under the time-concentration curve (AUC) with escalating dose of GO. There was a remarkable decrease in clearance with subsequent doses, as the half-life of GO was 62 hour (h) after the first 9 mg/m^2^ dose and 90 h after the second dose. Increase in Cmax was associated with a higher risk of hepatotoxicity, but no relationship between Cmax or AUC and complete remission (CR) rate was discovered. Therefore, responses to GO are not strictly dose dependent, particularly at high dose ranges [35].

### Clinical trials of GO in AML

GO received accelerated approval from FDA in 2000 based on the encouraging result of three open-label, multicenter, single arm phase II trials that enrolled 142 patients aged 60 or older in the first relapse of CD33+ AML treated with 9 mg/m^2^ of GO at 2-week intervals for 2 doses. There was a 30% overall response rate (ORR) with a 16.2% CR rate [36]. In a post-approval phase III trial (SWOG S0106; NCT00085709), newly diagnosed AML patients (aged 18 to 60 years) were randomized to receive either a single dose of GO 6 mg/m^2^ on day 4 in conjunction with the conventional ‘3 + 7’ DA induction chemotherapy (daunorubicin 45 mg/m^2^ on days 1–3 and cytarabine or AraC 100 mg/m^2^ on days 1–7, the DA regimen), or only DA but with 60 mg/m^2^ of daunorubicin (DNR) to balance the toxicity between groups. The addition of GO not only failed to improve CR rate, OS and event free survival (EFS) rate, but was associated with a higher early mortality during induction (5.5% vs 1.4%). Major causes of death were fatal hemorrhage and infection [37]. The safety and efficacy concern led to a quick withdrawal of GO from the market in 2010. A subsequent prospective randomized trial (NCRI AML17; ISRCTN55675535) revealed that when incorporating GO into the induction therapy of previously untreated AML, a single dose of 6 mg/m^2^ appeared not superior to a 3 mg/m^2^ dose with respect to clinical response and survival but was associated with an increased risk of hepatotoxicity and early mortality [38]. The GO doses selected by previous studies including SWOG S0106 aimed at saturating target CD33 antigen after a single dose. However pharmacokinetic studies have discovered a 90% saturation of CD33 even at a lower dose (3 mg/m^2^), and a rapid recycling and re-expression of CD33 on cell surface within 72 h after the first exposure to the drug, hence a lower but frequent dosing schedule was thought to be safer and more beneficial [39]. Over the past decade, several investigational studies using fractionated dosing schedule of GO were completed and were able to improve the safety profiles without compromising the clinical efficacy [21, 40, 41]. Based on these promising results, GO gained re-approval from FDA in September 2017 as monotherapy or in combination with conventional chemotherapy for the treatment of newly-diagnosed CD33+ AML and relapsed or refractory (R/R) CD33+ AML in adults [35, 42].

1. GO + chemotherapy as induction regimen for newly diagnosed AML

ALFA-0701 (registered with EudraCT, number 2007–002933-36) was one of the first phase III, open-label, randomized, multicenter trials evaluating GO administered in fractionated doses in conjunction with DA induction chemotherapy for newly diagnosed AML [40]. Two hundred seventy-one patients aged 50–70 with previously untreated AML were randomized to receive intensive doses of DA with or without GO 3 mg/m^2^ every 72 h on day 1, 4, and 7. Patients who achieved CR continued to undergo 2 cycles of consolidation therapy that consisted of intermediate doses of DA with or without GO 3 mg/m^2^ on day 1 of each of the 2 consolidation cycles in accordance with the initial randomization. GO recipients experienced a longer median EFS (17.3 vs 9.5 months), a higher 3-year EFS rate (39.8% vs 13.6%), and a longer median relapse free survival (RFS) (28 vs 11.4 months). Subgroup analysis showed that the clinical benefits on EFS and RFS were restricted to patients with favorable or intermediate-risk cytogenetics. The median OS was slightly improved in GO recipients according to the interim results, but the difference failed to reach statistical significance in the final updates of the study (27.5 vs 21.8 months, *p* = 0.16). No significant difference of CR or CR with incomplete platelet recovery (CR/CRi) after induction treatment was found between the GO and control arm (81% vs 75%, *p* = 0.25). Contrary to SWOG S0106, the number of early mortalities was similar [6 (4.6%) vs 5 (3.6%)] [40, 43]. A total of 85 patients in the study underwent AlloSCT, similar post-transplantation outcome and toxicity profile were observed in patients treated with and without GO [44]. Likewise, a meta-analysis of 3325 patients with newly diagnosed AML who were treated by GO + DA from 5 randomized controlled trials (RCT) (ALFA-0701, [40] SWOG S0106, [37] MRC AML15, [45] NCRI AML16, [23] and GOELAMS AML 2006 IR [46]) further confirmed that the addition of GO significantly reduced the risk of relapse and improved the 5-year OS. The survival benefit was also evident in patients with favorable or intermediate-risk cytogenetics, but not in those with high-risk cytogenetics. A lower incidence of early mortality was also identified in 3 mg/m^2^ dosed patients than 6 mg/m^2^ dosed patients [47]. A recent AML mouse model study suggested that the survival benefit seen with the combination of GO and DA might stem from an enhanced reduction of the leukemic stem cells (LSC) [48]. LSC was believed to be one of the leading causes of resistance to chemotherapy, persistence of measurable residual diseases (MRD) and relapse after CR [49].

Apart from GO + DA, various combination regimens have been studied or currently under development as frontline therapies for newly diagnosed AML (summarized in Table 1). There were attempts centered on modifying daunorubicin and cytarabine doses and adding new targeted agents or non- multidrug resistance (MDR) related drugs such as fludarabine. For example, in a recent phase II trial (NCT00909168), low dose GO (3 mg/m^2^ on day 6) with fludarabine, cytarabine, granulocyte colony stimulating factor and idarubicin (FLAG-Ida) were administered to 130 patients under 65 years old. 82% of patients achieved CR, and the 2-year OS and disease-free survival (DFS) were 63 and 54% respectively. 63.8% of patients underwent AlloSCT after the induction therapy [50]. GO was studied in the EORTC-GIMEM AML-17 trial, a randomized trial that evaluated the GO therapy followed by standard chemotherapy, MICE (mitoxantrone, cytarabine, etoposide) in older patients (age 61 to 75 years) with newly diagnosed AML [51]. In the arm with GO, two doses of GO (6 mg/m(2) on days 1 and 15) were given. For those patients in remission, two courses with or without GO (3 mg/m(2) on day 0) were given for consolidation. OS was the primary end point. In this study, 472 patients were enrolled. The ORR was similar (GO, 45%; no GO, 49%). However, the mortality rates were higher in the GO arm during induction and at day 60 (17% v 12 and 22% v 18%, respectively). Severe liver and hematologic toxicities were higher in the GO arm. Therefore, the sequential combination of GO and MICE chemotherapy had no survival benefit but worse toxicities for older patients with AML. For older patients who were ineligible for intensive chemotherapy, GO was proposed as a first-line monotherapy by a randomized phase III EORTC-GIMEMA AML-19 trial (NCT00091234). In this study, 237 patients aged above 60 were randomized to receive either GO (6 mg/m^2^ on day 1 and 3 mg/m^2^ on day 8) or best supportive care. GO recipients experienced a longer median OS (4.9 vs 3.6 months) and a higher 1-year OS rate (24.3% vs 9.7%), while presenting with similar rates of adverse events (AE). The clinical outcome was consistent across all genetic subgroups [21]. Nevertheless, GO + DA still appeared to be the regimen that generated the best clinical outcome so far. In a recent meta-analysis, GO + DA was compared to most other induction therapy agents for newly diagnosed AML. GO + DA was associated with significantly longer OS and RFS compared with most evaluated regimens, but a higher rate of hemorrhage and hepatotoxicity [52].

2.) GO +/− chemotherapy as re-induction regimen for relapsed or refractory AML

**Table 1:** Reported clinical trials of gemtuzumab ozogamicin for acute myeloid leukemia

Trial name or NCT number (reference)	Phase	Age(number)	ND or R/R AML	Intervention	CR/CRi (CR)	DFS/EFS/RFS	OS	Early death (< 30 days)	Severe adverse events (Grade ≥ 3)
[37]	III	18–60 (595)	ND	GO (6 mg/m^2^ D4) + DA (3 + 7)	76% (69%)	5-yr: 43%	5-yr: 46%	5%	Grade 4 thrombocytopenia (48%), grade 4 or fatal nonhematologic induction toxicity (21%)
ALFA-0701 [43]	III	50–70 (271)	ND	GO (3 mg/m^2^ D1, 4, 7) + DA (3 + 7)	81% (70%)	17.3 mo 2-yr: 42% 3-yr: 40%	28 mo 5-yr: 41%	3.8%	Infection (78%), hemorrhage (23%), VOD (3.7%)
MRC AML15 [45]	III	0–71 (1113)	ND	GO (3 mg/m^2^ D1) + 2 cycles of DA, FLAG-Ida, or ADE	85% (82%)	5-yr: 39%	5-yr: 43%	11%	Myelosuppression (thrombocytopenia and neutropenia with unknown incidence)
NCRI AML16 [23]	III	51–84 (1115)	ND	GO (3 mg/m^2^ D1) + DA (3 + 10 then 3 + 8) or DC (daunorubicin D1, 3, 5 + clofarabine D1–5)	69% (60%)	3-yr: 21%	2-yr: 35% 3-yr: 25%	9%	Gastrointestinal events (19%), liver chemistry abnormalities (17%)
NCRI AML17 [38]	III	0–81 (788)	ND	GO (3 or 6 mg/m^2^ D1) + DA (3 + 10) or ADE (10 + 3 + 5);	3 mg GO: 89% (82%) 6 mg GO: 86% (76%)	3 mg GO: 4-yr 44% 6 mg GO: 4-yr 38%	3 mg GO: 4-yr 50% 6 mg GO: 4-yr 47%	3 mg GO: 3% 6 mg GO: 7%	3 mg vs 6 mg GO: VOD (0.5% vs 5.6%), increased ALT (17% vs 7%)
GOELAMS AML 2006 IR [46]	III	18–60 (238)	ND	GO (6 mg/m^2^ D1) + DA (3 + 7)	CR 91%	51%	53%	10%	Hepatotoxicity (23%), severe VOD 4 cases
NCT00909168 [50]	II	18–65 (130)	ND	GO (3 mg/m^2^ D6) + FLAG-Ida (fludarabine 30 mg/m^2^ and cytarabine 2 mg/m^2^ D1–5, idarubicin 10 mg/m^2^ D1, 3, 5)	85% (82%)	1-yr: 77% 2-yr: 58% 5-yr: 52%	1-yr: 80% 2-yr: 63% 5-yr: 62%	3%	AEs of all grades: fever of unknown origin (52%), bacteremia (26%), HSV infection (18%), pneumonia (17%), mucositis (17%)
EORTC-GIMEMA AML-17 [51]	III	61–75 (472)	ND	GO (6 mg/m^2^ D1, 15) + MICE (mitoxantrone 7 mg/m^2^ D1, 3, 5; etoposide 100 mg/m^2^ D1–3; and cytarabine 100 mg/m^2^ D1–7)	45% (36%)	–	7.1 mo	17%	Infection (37%), neutropenic fever (26%), hepatotoxicity (15%), bleeding (11%)
EORTC-GIMEMA AML-19 [21]	III	61–75 (237)	ND, not fit for chemotherapy	GO (6 mg/m^2^ D1 + 3 mg/m^2^ D8)	27% (15%)	–	4.9 mo, 1-yr: 24%	11%	Infections (35%), febrile neutropenia (18%), bleeding (13%)
MyloFrance 1 [41]	II	22–80 (57)	First relapse	GO (3 mg/m^2^ D1, 4, 7)	33% (26%)	11 mo	8.4 mo	7%	Sepsis (31.5%), fever (15.8%), rash (10.5%), pneumonia (7%), bleeding (7%)
NCT00143975 [54]	II	18–60 (93)	Refractory to 1 cycle of induction	GO (1 mg/m^2^ D1) + ATRA (45 mg/m^2^ D4–6, 15 mg/m^2^ D7–28) + cytarabine (3 mg/m^2^/12h D1–3) + mitoxantrone (12 mg/m^2^ D2–3)	51% (30%)	–	16 mo 4-yr: 32%	3%	Septicemia (46%), pneumonia (22%), gastrointestinal events (15%)
NCT00766116 [55]	I/II	29–82 (50)	R/R	GO (6 mg/m^2^ D7, 21) + Azacytidine (75 mg/m^2^ D1–6)	Phase I: 50% (25%) Phase II: 24% (11%)	–	–	0	Febrile neutropenia (75%), infections (17%)
NCT00895934 [56]	I/II	50–79 (52)	R/R	GO (3 mg/m^2^ D4, 8) + azacytidine (75 mg/m^2^ D1–7) + vorinostat (400 mg D1–9)	Phase I: 40% Phase II: 42% (21%)	–	–	7%	Febrile neutropenia (75%), infections (31%)
NCT00882102 (Daver et al. 2016) [57]	II	27–89 (110)	ND and R/R	GO (3 mg/m^2^ D5) + decitabine (20 mg/m^2^ D1–5) q14d	35%	ND: 7 mo R/R: 1 mo	ND: 7 mo R/R: 3.5 mo	–	Febrile neutropenia (45%), infections (21%)
NCT00801489 [61]	II	19–76 (45)	ND CBF AML	GO (3 mg/m^2^ D1) + FLAG (fludarabine 30 mg/m^2^ and cytarabine 2 mg/m^2^ D1–5, G-CSF 5 μg/kg D1)	95% (91%)	3-yr: 85%	3-yr: 78%	4%	Fever of unknown origin (24%), pneumonia (18%), sepsis (10%), transaminase elevations (8%)

*ADE* Cytarabine, daunorubicin, and etoposide, *ALT* Alanine transaminase, *CBF* Core-binding factor, *D* Day, *DA* Daunorubicin + cytarabine, *DC* Daunorubicin + clofarabine, *DFS/EFS/RFS* Disease-, event-, or relapse- free survival, *FLAG-Ida* Fludarabine, cytarabine, granulocyte colony-stimulating factor, and idarubicin, *GO* Gemtuzumab ozogamicin, *mo* Month, *ND* Newly diagnosed, *OS* Overall survival, *RFS* Relapse-free survival, *R/R* Refractory or relapse, *yr* year

In the relapsed setting, GO was also effective and safe according to MyloFrance 1, a single-arm phase II trial. In this study, fractionated GO (3 mg/m^2^ on days 1, 4 and 7) was administered to 57 patients with AML in their first relapse. 15 (26%) patients achieved CR. The median RFS was 11 months. No severe hepatotoxicity was discovered [41]. Several options of combination therapy have also been proposed for R/R AML (Table 1). Salvage therapy with fractionated GO + intermediate-dose DA was retrospectively analyzed in 36 high-risk AML patients (median age 54 years) with short CR1 duration (< 6 months) or primary refractory disease. The treatment produced a 38.8% ORR with a 22.2% CR rate, a 26% 2-year OS, and a 18.5% 2-year RFS [24]. A similar study conducted in 24 high-risk AML patients achieved 50% CR. 1-year OS was 50.7%. Thirteen patients went on to AlloSCT. Subgroup analysis revealed that the survival was longer for those who received reduced intensity conditioning compared to those undergoing myeloablative conditioning regimen. Therefore, fractionated GO + intermediate-dose DA might be considered as a potential bridge therapy to transplantation while downgrading the toxicity of transplantation regimen [53]. Another effective and tolerable salvage and bridge therapy combination was GO (3 mg/m^2^ on day 1) plus all-trans retinoic acid, high-dose cytarabine and mitoxantrone. The regimen was studied in a phase II trial (NCT00143975) that enrolled 93 patients aged 18–60 years refractory to one cycle of induction therapy. 57 (61.5%) patients achieved ORR including 47 (51%) CR. Among them, 51 patients underwent AlloSCT and had a 4-year OS rate of 49% [54]. For patients who were unable to tolerate intensive chemotherapies, HMAs appeared to be appropriate alternatives. GO was administered in conjunction with azacytidine in a phase I/II trial (NCT00766116). In the updated report of 50 evaluable patients, 12 (24%) achieved CR/CRi. A concurrent in vitro study discovered that azacytidine-pretreated AML cells exhibited increased response to GO treatment [55]. In another phase I/II trial (NCT00895934), vorinostat, a histone deacetylase inhibitor, was added to the regimen of GO + azacytidine. 18 (41.9%) out of the 43 evaluable patients achieved CR/CRi with a median OS of 7.5 months [56]. In addition, decitabine was also evaluated together with GO in older patients with newly diagnosed or R/R AML in a phase II trial (NCT00882102). In the subgroup of R/R AML within 1-year remission, the CR/CRi rate was 18% and the median OS was 3.5 months, while in the subgroup of newly diagnosed AML, the CR/CRi rate was 45% and the median OS was 7 months. Therefore, GO + decitabine was a good option for patients who are not suitable for intensive chemotherapy [57]. For patients suffering from relapse after AlloSCT, a 4-day course of 1.0 g/m^2^ ARA-C followed by 1-day of 9 mg/m2 GO was reported in one retrospective study. The regimen provided a short-term disease control (ORR 60%, median OS 103 days, median EFS 76 days) with manageable toxicities [58]. A number of ongoing trials of GO in AML patients were listed in Table 2.

**Table 2:** Ongoing clinical trials of gemtuzumab ozogamicin for acute myeloid leukemia

NCT number	Phase	Conditions	Interventions	Recruitment
NCT03727750	IV	CD33+ R/R AML	Evaluate the QTc, pharmacokinetics, safety of GO	Not yet recruiting
NCT03374332	II	R/R AML	GO (D1, 4, 7) followed by non-engraftment donor leukocyte infusion	Not yet recruiting
NCT03737955	II	AML with MRD	GO (D1, 4, 7)	Recruiting
NCT02473146	II/III	Elderly AML patients	GO (D1, 4) + cytarabine (D1–7) vs idarubicin (D1–3) + cytarabine (D1–7)	Recruiting
NCT03672539	II	R/R AML	GO + CPX-351 (liposome-encapsulated daunorubicin-cytarabine)	Recruiting
NCT02221310	II	High risk CD33+ AML/MDS	GO + busulfan + cyclophosphamide followed by ASCT	Recruiting
NCT03839446	II	AML refractory to initial standard induction	GO + mitoxantrone + etoposide	Recruiting
NCT02117297	II	Average risk AML/MDS	ASCT followed by GO (D3, 56 post transplatation)	Recruiting
NCT03848754	I	R/R AML	GO (D1, 4, 7) + Pracinostat	Recruiting
NCT03531918	I/II	ND AML or high-grade myeloid neoplasm	GO + G-CSF + Cladribine + Cytarabine + Mitoxantrone	Recruiting
NCT03900949	I	ND FLT-3 mutated AML	GO (D1, 4, 7) + DA (3 + 7) + midostaurin (D8–21)	Recruiting
NCT00801489	II	ND AML and high-risk MDS	GO + fludarabine phosphate + cytarabine + filgrastim-sndz + idarubicin hydrochloride	Recruiting
NCT03390296	I/II	R/R AML	GO + azacytidine + venetoclax or avelumab	Recruiting
NCT01409161	II	ND acute promyelocytic leukemia	Tretinoin + Arsenic Trioxide ± GO	Recruiting

*GO* Gemtuzumab ozogamicin, *ASCT* Allogenic stem cell transplantation, *DA* Daunorubicin + cytarabine, *G-CSF* Granulocyte colony stimulating factor, *MDS* Myelodysplastic syndrome, *MRD* Measurable residual disease, *ND* Newly diagnosed, *R/R* Refractory or relapse

### Factors affecting the response to GO

It is important to note that the clinical benefits of adding GO to standard induction regimen on EFS and RFS were restricted to patients with favorable or intermediate-risk cytogenetics [40, 43]. Aside from cytogenetics, CD33 was another independent biomarker predicting the clinical outcome of GO treatment for adult AML patients. Patients with higher expression of CD33 generally derived the most benefit from GO [59, 60]. *NPM1* and *FLT3*-*ITD* mutations often increased in prevalence with high CD33 levels, but their impact on the effect of GO varied between studies [40, 47]. Although low expression of CD33 was less likely to benefit from GO, a subgroup of patients with core-binding factor (CBF)-AML showed dramatic response to GO [22]. However, it is important to note that CBF-AML can respond well to high-dose induction regimen alone. In one study (NCT00801489), 45 patients with CBF-AML who received GO in combination with FLAG (FLAG-GO) as a front-line therapy achieved a 75% ORR, with a 3-year OS and RFS of 78 and 85% respectively [61]. Encouraging response and outcome were also seen in CBF-AML patients treated with FLAG-Ida + GO [62]. Single nucleotide polymorphism (SNP) in CD33 gene appears to play a role in AML responses to GO. One typical example was rs12459419 C > T in the splice enhancer region which produces a truncated form of CD33. It has been reported that patients aged 0–29 with CC genotype, but not CT or TT genotype, for rs12459419 showed a substantial response to GO with low relapse risk and high DFS [63]. However uncertainties about the role of rs12459419 genotype in GO-based therapy still existed since similar correlation failed to be reproduced by either in vitro studies or a subsequent study conducted in young adults aged 13–69 [64–66]. New SNPs of other biomarkers are still being explored. Recently rs1045642 C > T of *ABCB1* encoding the drug-transporter PgP-1 was shown to correlate with a better outcome in patients with a CT/TT genotype compared to CC genotype [67]. In addition, it has been reported that higher expression of ATP binding cassette transporter 3 (ABCA3) and mutation in *HFE* gene encoding human hemochromatosis protein both independently predicted resistance to the treatment of fractionated GO plus intensive chemotherapy. This was attributed to impaired CD33 internalization and drug sequestration in lysosomes or endosomes [68, 69]. Another recent study discovered that PP242, a mTORC1/2 kinase inhibitor, was able to potentiate the cytotoxicity of GO by inducing lysosomal activation and progression of cell cycle, which gives new insights into optimizing the response to GO [70].

### Safety and toxicity

The most common toxicity of GO was myelosuppression, notably persistent thrombocytopenia and neutropenia. According to ALFA-0701, grade ≥ 3 hemorrhage due to a longer duration of treatment-induced thrombocytopenia was more frequently encountered among GO + DA recipients compared to DA-only recipients [30 (22.9%) vs. 13 (9.5%)]. The average time to platelet count recovery was significantly longer in GO-treated patients (25 vs 21 days). Infections associated with febrile neutropenia represented one of the major causes of early death. But the incidence was similar between patients treated with or without GO [102 (77.9%) vs 106 (77.4%)], so was the median time to neutrophil count recovery (22 vs 22 days). Frequent monitoring and treatment interruption were often required when severe hematologic events occurred [43]. Another drug-specific AE related to GO therapy was hepatotoxicity, including the development of veno-occlusive disease (VOD), also known as sinusoidal obstruction syndrome (SOS), especially after AlloSCT. But lower GO dose (3 mg/m^2^) with a fractionated regimen significantly reduced the risk of VOD. As reported in ALFA-0701, the incidence of VOD did not significantly differ between the GO and control arm [6 (4.6%) vs 2 (1.5%), *p* = 0.165], nor did the result of liver chemistry abnormalities [43]. In another study, out of 146 patients undergoing AlloSCT previously treated with GO, 11 (8%) developed VOD after transplantation, with death in 3 patients, comparable to the incidence of historical cohorts of patients not receiving GO [71]. Prophylaxis of VOD for high-risk patients with agents such as recombinant human soluble thrombomodulin might further lower the risk [72]. Currently a phase IV study has been initiated aiming at providing more detailed information about the pharmacokinetics and safety of GO (NCT03727750).

## Novel ADCs under development for AML

Several novel ADCs featured by modified structure or new target antigens are currently under development for AML (Table 3).

**Table 3:** Novel antibody-drug conjugates for acute myeloid leukemia

Drug name	Target	Payload	Linker	Development stage
Vadastuximab talirine (SGN-CD33A)	CD33	PBD dimer	Dipeptide linker (protease-cleavable)	Phase III
IMGN779	CD33	DGN462	Disulfide linker	Phase I
AVE9633 (huMy9–6-DM4)	CD33	DM4	Disulfide linker	Phase I (terminated)
IMGN632	CD123	IGN	Dipeptide linker (protease-cleavable)	Phase I
SGN-CD123A	CD123	PBD dimer	Dipeptide linker (protease-cleavable)	Phase I (terminated)
CLT030	CLL-1	IQB	Dipeptide linker (protease-cleavable)	Preclinical
Anti-CLL-1 ADC	CLL-1	PBD dimer	Disulfide linker	Preclinical

*DM4* N20-deacetyl-N20-(4-mercapto-4-methyl-1-oxopentyl)maytansine, *IGN* Indolinobenzodiazepine pseudodimer, *IQB* Isoquinolidinobenzodiazepine, *PBD* Pyrrolobenzodiazepine

### Anti-CD33 ADCs

#### Vadastuximab talirine (SGN-CD33A)

SGN-CD33A is a novel anti-CD33 mAb conjugated to 2 molecules of pyrrolobenzodiazepine (PBD) dimers via a protease-cleavable maleimidocaproyl-valinealanine dipeptide linker on engineered cysteine residues. The engineering technique creates a highly homogenous ADC with a controlled drug-antibody ratio. SGN-CD33A is highly stable in circulation with relatively less off-target toxicity compared to GO. PBD dimer damages DNA by inducing DNA crosslinking after binding to the minor groove [73].

Single-agent SGN-CD33A demonstrated efficacy and a tolerable toxicity profile for AML. In a dose-escalation phase I trial (NCT01902329), 40 μg/kg was identified as the recommended phase 2 dose (RP2D). 14 (54%) out of 27 elderly patients with previously untreated AML achieved CR/CRi [74]. In another dose-escalation phase I trial (NCT01902329), 131 AML patients (aged 73 on average) received either a single dose (range 5–60 μg/kg) of SGNCD33A on day 1 or a dose of 20 μg/kg on days 1 and 4 of a 21-day treatment cycle for a maximum of 2 cycles. This study also defined a single intravenous dose of 40 μg/kg on day 1 as RP2D. At this dose, 5 (28%) out of 18 patients obtained CR/CRi, with bone marrow blast clearance observed in 8 (44%) patients. Additionally, subgroup analysis showed that 15 (56%) out of the 27 patients with previously untreated AML achieved CR/CRi with bone marrow blast clearance observed in 19 (70%) patients. Overall, there was an 8% 30-day mortality rate. Myelosuppression was the most common AE. Median time to full count recovery was 6.4 weeks for neutrophils (≥1000/μL) and 10.6 weeks for platelets (≥100,000/μL) in patients across all dose levels who achieved CR/CRi. Other AEs included fatigue, nausea, and diarrhea. No significant risk of VOD was reported [75].

SGN-CD33A was explored in combination with the ‘3 + 7’ DA induction chemotherapy in 42 patients with newly diagnosed AML in a phase I trial (NCT02326584). The recommended SGN-CD33A dosing schedule in this combination regimen was 20 μg/kg on day 1 and 10 μg/kg on day 4. The CR/CRi rate was 78%. 74% of the CR/Cri patients achieved negative MRD. All patients experienced grade ≥ 3 myelosuppression with a median time to neutrophil and platelet count recovery of 4.7 and 5 weeks respectively. No 30-day mortality or significant hepatotoxicity was observed [76]. In another study, HMA was reported to be able to enhance the cytotoxicity of SGN-CD33A by facilitating DNA binding of PBD and upregulating CD33 expression [77]. In this study, a 10 μg/kg dose of SGN-CD33A was added every 4 weeks on the last day of a HMA regimen (either a 7-day regimen of azacitidine or a 5-day regimen of decitabine) for a median treatment duration of 19.3 weeks in 53 newly diagnosed AML patients (average age 75) who were unfit for or declined intensive chemotherapy in a phase I trial (NCT01902329). 70% of patients experienced CR/CRi with a 74% blast clearance rate and a median OS and RFS of 11.3 and 7.7 months respectively. Similar activity was observed across both low- and high-risk sub-populations. The response rate appeared to be increased as compared to the historical data of HMA monotherapy, but there was also a higher risk of grade ≥ 3 myelosuppression. Median time to neutrophil and platelet count recovery was 10.6 and 10.1 weeks respectively. The 30-day mortality rate was 2% [78]. This has led to a phase III trial (CASCADE, NCT02785900) comparing HMA with or without SGN-CD33A in elderly patients with newly diagnosed AML. However, the study was prematurely terminated because of a high rate of death including fatal infections in the SGN-CD33A containing group.

In the post-remission setting, SGN-CD33A combined with high-dose cytarabine seemed to provide an effective consolidation therapy according to a phase 1b dose-escalation study (NCT02326584). GO was given on day 1 of a 28-day cycle for a median of 2 cycles in combination with high-dose cytarabine. Twenty microgram per kilogram was identified as the MTD. Of the 19 evaluable patients in their first remission, 15 (79%) maintained remission, and 18 (95%) patients were alive, and 9 patients (43%) went on to receive AlloSCT. All patients experienced grade ≥ 3 myelosuppression, but no 60-day mortality was detected. The same study also evaluated another group of patients in remission and had completed planned post-remission therapies including AlloSCT. Five microgram per kilogram of GO was administered as a single agent on day 1 of a 6-week cycle for a median of 3 cycles. 15 (75%) out of the 20 evaluable patients were able to maintain remission, suggesting single-agent SGN-CD33A to be a good option for AML maintenance therapy. There was no dose limiting toxicity (DLT). Myelosuppression and mild non-hematologic toxicities were reported but were generally manageable [79]. Further randomized large clinical trials are warranted to confirm these results.

### IMGN779

IMGN779 comprises a humanized anti-CD33 mAb conjugated via a cleavable disulfide linker to DGN462, a novel DNA-alkylating agent. Preclinical studies revealed that IMGN779 was highly active against AML cell lines and LSCs both in vitro and in vivo, especially cells from patients with *FLT3-ITD* mutation while sparing normal hematopoietic stem cells (HSC), suggesting a potentially lower risk of myelosuppression [80, 81]. There were also studies showing that combining IMGN779 with other agents such as cytarabine or olaparib, a PARP Inhibitor, significantly enhanced anti-tumor activity in preclinical models of AML [82, 83]. The promising results of preclinical studies have led to an ongoing dose-escalation phase I trial (NCT02674763) in 50 patients with R/R CD33+ AML. IMGN779 was administered in either every 2-week (36 patients; dose range: 0.39–1.5 mg/kg) or a weekly (14 patients; dose range: 0.39–0.54 mg/kg) schedule for a 28-day cycle. Interim results showed that the most frequent SAEs were febrile neutropenia (40%), bacteremia (22%), and pneumonia (20%). No DLT or drug-related deaths have been observed in either schedule. Overall, 11 (41%) out of 27 patients with measurable circulating blasts experienced a > 30% reduction in bone marrow blasts. Further escalation of the dosing schedules is currently ongoing to optimize the clinical response [84, 85].

### AVE9633 (huMy9–6-DM4)

AVE9633 is a humanized anti-CD33 IgG1 mAb conjugated to DM4 via a disulfide linker. DM4 is a maytansinoid derivative that binds to tubulin and impede microtubule assembly, leading to G2/M cell cycle arrest and subsequent cell apoptosis. Several phase I trials have been conducted to test AVE9633 in adult patients with R/R AML. Even though no significant risk of myelosuppression was observed, no obvious clinical efficacy was noted since only two CR/CRi were reported out of 54 patients [86, 87]. No further clinical trials have been initiated so far.

### Anti-CD123 ADCs

CD123 is the α chain of the interleukin-3 receptor (IL-3R), which forms a heterodimer with the β subunit of the IL-3R [88]. CD123 is expressed on most AML tumor cells at a higher level compared to normal hematopoietic progenitors [89]. It has also been found selectively expressed on LSCs [90]. Therefore, CD123 represents a promising target for AML. One CD123-targeted agent that entered phase II development was SL-401, a recombinant fusion protein composed of a human IL-3 and a truncated diphtheria toxin [91]. Interim result from an ongoing phase I/II trial (NCT02270463) conducted in high-risk AML patients in remission showed that out of 7 MRD+ patients who received SL-401 at recommended doses, 1 subsequently went on to AlloSCT, and 2 remained in remission at 3+ and 4+ months. SL-401 was well tolerated [92]. Several ADCs targeting CD123 are in clinical development and detailed as the following.

#### IMGN632

IMGN632 comprises a humanized anti-CD123 mAb conjugated, via a dipeptide linker, to an indolinobenzodiazepine pseudodimer (IGN) class of cytotoxic payload, a newly developed DNA-alkylating agent. IMGN632 demonstrated an abundant antitumor activity against AML cell lines with or without poor prognostic biomarkers (FLT3-ITD, MDR1, TP53, etc.) and in multiple AML xenograft models, including one model which appeared to be resistant to azacitidine and cytarabine [93, 94]. One study evaluated the effect of combination of GO and IMGN632 on bone marrow cells from 17 AML patients. The bone marrow leukemia cells from all the 17 patients were highly sensitive to IMGN632 at a concentration 150-fold lower than the one at which normal progenitors were affected, whereas only 6 out of 17 patient samples were sensitive to GO at the concentration that did not impact normal progenitors, suggesting that IMGN632 has a superior therapeutic window over GO in AML and might be a novel CD123 targeted ADC with limited myelosuppression [93]. IMGN632 was studied in combination with a PARP inhibitor in a preclinical model and the combination synergistically enhanced the anti-leukemic effect [95].

Given the encouraging result of preclinical and early clinical studies, phase I trials of IMGN632 (NCT03386513) are initiated in patients with R/R AML and other CD123+ hematologic malignancies. Interim results showed that among 12 evaluable patients with R/R AML, 4 (33%) achieved CR/CRi. No DLTs have been noted at doses up to 0.18 mg/kg. The most frequently encountered treatment-emergent AEs (TEAE) of any grades were primarily gastrointestinal (diarrhea, nausea; 25–42%), hematologic (febrile neutropenia; 42%), or vascular (peripheral edema, hypotension, sinus tachycardia; 25–33%). The most common SAEs were febrile neutropenia (42%) and lung infection (25%), but none of these events were considered related to IMGN632 and no discontinuation of therapy due to AEs have occurred [96].

#### SGN-CD123A

SGN-CD123A is composed of a humanized anti-CD123 mAb with a PBD dimer attaching to an engineered cysteine residue on each one of the two heavy chains via a protease-cleavable dipeptide linker. Preclinical studies showed that SGN-CD123A demonstrated anti-tumor activities against AML cell lines and primary samples from AML patients with or without adverse cytogenetic profiles or *FLT3* mutations. SGN-123A induced durable CR in multiple AML xenograft models [97, 98]. However the first in-human phase I trial (NCT02848248) was terminated in May 2018 because of safety concerns in AML patients.

### Anti-CLL1 ADCs

Human C-type lectin-like molecule-1 (CLL-1), also known as CLEC12A or MICL, is a transmembrane glycoprotein, highly expressed on AML blast cells and LSCs. Different from CD33 and CD123, CLL-1 is completely absent on normal HSCs, making it an ideal therapeutic target for AML [99]. CD3/CLL-1 bispecific IgG antibody (MCLA-117) is currently under a phase I study for patients with newly diagnosed or R/R AML (NCT03038230) [100]. CD123/CLL-1 chimeric antigen receptor (CAR)-T cells have reached phase III development for patients with R/R AML (NCT03631576) [101].

The first anti-CLL-1 ADC contained a PBD dimer conjugated via a disulfide linker. It demonstrated robust activities at depleting tumor cells in AML xenograft models. In a study of cynomolgus monkey model, neutropenia was the major DLT. No obvious off-target toxicity was observed at the dose that can deplete neutrophils and monocytes [102]. CLT030 is an ADC with a humanized anti-CLL1 mAb site-specifically conjugated to two molecules of isoquinolidinobenzodiazepine (IQB), a novel DNA cross-linker, through a cleavable dipeptide linker. CLT030 demonstrated robust activities in inhibiting in vitro LSC colony formation and reducing in vivo tumor cell survival in AML xenograft models. CLT030 had only limited effect on the normal differentiation of healthy HSCs into various lineages [103]. Clinical studies of anti-CLL-1 ADCs are yet to be done.

### Considerations in using GO for AML therapy

GO as the first approved CD33-directed ADC is indicated for the treatment of newly-diagnosed CD33+ (> = 1%) adult AML patients as well as for relapsed or refractory CD33+ pediatric (2 years and older) and adult AML patients. There have been several newly approved agents and regimens for AML therapy in the past 2 years [104]. A variety of factors should be considered for selection of an induction regimen, such as age (younger or older than 60), co-morbidities (cardiac functions as well as other vital organ functions), risk stratifications including cytogenetic abnormalities, genetic mutations such as the targetable mutations FLT3+, IDH1+, or IDH2+. In addition, it is also important to know whether the AML has any myelodysplasia-related changes or is therapy-related. Since one particular adverse event associated with GO is liver function abnormality /VOD, it is important to pay attention to liver toxic medications, and hepatitis history.

## Conclusion

GO is the first ADC approved by FDA for induction therapy of AML. No randomized data are available yet supporting the addition of GO in consolidation or maintenance therapy. Optimal dose and schedule of GO in combination regimens are still being determined. New ADCs targeting CD33, CD123 and CLL-1 (SGN-CD33A, IMGN779, IMGN632, CLT030) are under active development. It is foreseeable that the treatment landscape of AML will likely continue to expand and evolve with more ADCs being developed and approved.

## Data Availability

The material supporting the conclusion of this review has been included within the article.

## Abbreviations

ADC: Antibody-drug conjugate
DLT: Dose-limiting toxicity
MTD: Maximal tolerated dose
